# Mucopolysaccharidosis Type I Presenting with Persistent Neonatal Respiratory Distress: A Case Report

**DOI:** 10.3390/diseases11020067

**Published:** 2023-04-28

**Authors:** Ali Alsuheel Asseri, Ahmad Alzoani, Abdulwahab M. Almazkary, Nisreen Abdulaziz, Mufareh H. Almazkary, Samy Ailan Alahmari, Arul J. Duraisamy, Shruti Sureshkumar

**Affiliations:** 1 Department of Child Health, King Khalid University, Abha 62529, Saudi Arabia; 2 Department of Neonatology, Abha Maternity and Children Hospital, Ministry of Health, Abha 62521, Saudi Arabia; alzoaniasiri44@hotmail.com (A.A.); nabdelmagid@moh.gov.sa (N.A.); malmazkary@moh.gov.sa (M.H.A.); sealahmari@moh.gov.sa (S.A.A.); 3 College of Medicine, King Khalid University, Abha 62529, Saudi Arabia; 438801388@kku.edu.sa; 4 PerkinElmer Health Sciences Pvt Ltd., Tharamani 600113, India; arul.duraisamy@perkinelmer.com (A.J.D.); shruti.sureshkumar@perkinelmer.com (S.S.)

**Keywords:** MPS I, neonatal respiratory distress, pulmonary surfactant dysfunction, α-L-iduronidase

## Abstract

Mucopolysaccharidosis type I (MPS I) is a rare inherited autosomal recessive lysosomal storage disorder. Despite several reports on MPS I-related neonatal interstitial lung disease, it is still considered to be an under-recognized disease manifestation. Thus, further study of MPS I is required to improve specific therapies and management strategies. The current report describes a late preterm baby (36 weeks gestational age) with neonatal onset of interstitial lung disease eventually diagnosed as MPS I. The neonate required prolonged respiratory support and oxygen supplementation that further escalated the likely diagnosis of inherited disorders of pulmonary surfactant dysfunction. Whole-exome sequencing confirmed the diagnosis of MPS I, following the observation of low levels of the enzyme α-L-iduronidase. The results highlight the necessity of considering MPS I-related pulmonary involvement in newborns with persistent respiratory insufficiency.

## 1. Introduction

Mucopolysaccharidoses (MPSs) are a rare disorder of the lysosomes, which occur due to an inherited deficiency of an enzyme involved in the degradation of glycosaminoglycans (GAGs). With the exception of mucopolysaccharidosis type II, which is X-linked, these disorders are autosomal-recessive [1]. There are currently thirteen MPSs that have been identified, and two of them were found in the 2020s [1]. Mucopolysaccharidosis type I (MPS I) is a rare inherited autosomal recessive lysosomal storage disorder caused by pathogenic α-L-iduronidase (IDUA) gene variant mutations, resulting in a deficiency of IDUA activity [1,2]. The IDUA gene has 14 exons and 13 introns and is found on chromosome 4 at location 4p16.3 [1,2,3,4]. There have been >300 reported variants of IDUA in the Human Genetic Mutation Database, the majority of which are missense/nonsense mutations [2]. Several studies have reported that p.W402X, p.Q70X, p.P533R, and p.G51D are the most common IDUA disease-causing mutations [2,4,5,6]. Furthermore, IDUA mutations are classified according to the disease phenotype, from severe to attenuated phenotypes [2,4]. Clarke et al. [4] performed a detailed genotype and phenotype correlation analysis based on data from a registry of >500 patients, and concluded that certain genotypes are predictive of severe and attenuated phenotypes. They highlighted the need for further study of potential biomarkers to assist in the prediction of the phenotype of patients and to further guide therapies.

The global prevalence of MPS I is estimated to be 1 in 100,000 births [7,8]. Compared to the published rates from Europe and the United States, the prevalence of MPS I has been found to be greater in the Middle East, particularly Saudi Arabia [9]. Based on a retrospective analysis of data from different centers in Saudi Arabia, the estimated incidence of MPS I is between 2 and 4 cases per 100,000 live births [9,10,11]. However, nationwide incidence studies are recommended for real estimates of the incidence of MPS I.

Deficiency in IDUA enzyme activity leads to impaired GAG degradation and subsequent pathologic accumulation in the cellular lysosomes of various body tissues [8,12]. Hurler, Hurler–Scheie, and Scheie syndromes are the three clinically categorized phenotypes of MPS I. These classifications are based on the age of onset and the rapidity of disease progression. Although patients with MPS I appear normal at birth, due to its progressive nature, the signs and symptoms usually become apparent during early childhood [2,4,5,6,7,8,9].

MPS I can present with a wide spectrum of severity. The most commonly observed clinical symptoms include coarse facial features, growth impairment, joint contractures, neurologic abnormalities, and upper airway obstructive features, followed by progressive cardiovascular complications [8,12,13,14]. Deposition of GAG in the lower respiratory system is considered a rare manifestation of MPS I and may cause tracheobronchomalacia, disruption of airway clearance, and early neonatal respiratory distress [13,14]. There are several reports of MPS I-related neonatal interstitial lung disease; however, MPS I is still an under-investigated disease, with severe clinical manifestations that require further study to improve early diagnosis and identify novel specific therapies for case management [13,15]. Here, the case of a neonate with genetically confirmed MPS I who presented with early neonatal interstitial lung disease is described. To the best of our knowledge, this is the first report of MPS I-related interstitial lung disease from Saudi Arabia.

## 2. Case Presentation

A late preterm male neonate was delivered by cesarean section at 36 weeks of gestation to a 34-year-old mother. She had a history of two previous deliveries by cesarean section. The mother had an uneventful pregnancy. The neonate was born to a second-degree consanguineously married couple with no significant family history. The neonate, weighing 3400 g, cried immediately after birth with an APGAR score of 6 and 8 at 1 and 5 min, respectively. He was admitted to the newborn nursery for observation. During the nursery observation, the newborn exhibited labored breathing with chest retractions and cyanosis. The newborn was subsequently transferred to the neonatal intensive care unit for further management. Arterial blood gas analysis showed saturation of 60%, pH of 7.22, pO_2_ of 65 mmHg (normal 75–100 mmHg), bicarbonate levels of 18 mEq/L (normal 22–29 mEq/L), and pCO_2_ of 48 mmHg (normal 35–45 mmHg). In addition, the results of the laboratory tests were as follows: hemoglobin level, 18 g/dL (reference range (R.R.), 14–24 g/dL); mean corpuscular volume, 69.4 fl (R.R., 96.0–108.0 fl); mean corpuscular hemoglobin level, 19.6 pg (R.R., 32.0–34.0 pg); hematocrit, 52. The white blood cell and platelet counts were within normal limits. The C-reactive protein levels were 25 mg/L (normal <10 mg/L), and the erythrocyte sedimentation rate was 35 mm/h (R.R., 0–20 mm/h). The electrolyte levels, including potassium, sodium, glucose, and calcium, as well as kidney and liver function, were normal. Kidney function was assessed by the serum creatinine level, which was within normal range for age. The liver enzymes (alanine transaminase and aspartate transaminase), serum albumin, and prothrombin time were within normal range.

**Figure 1:**
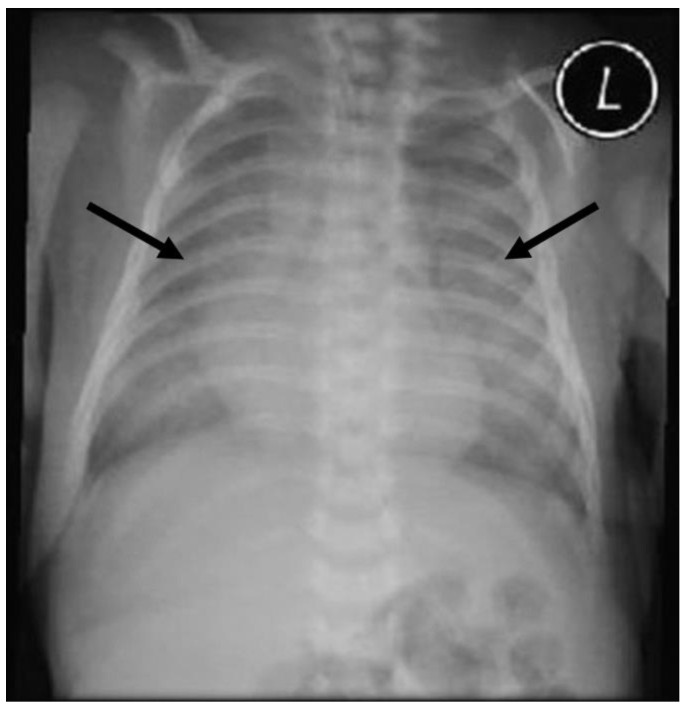
Chest radiography (CXR) (2 h after birth) revealed diffuse bilateral lung ground-glass opacities with air bronchograms (arrows); L = left.

The baby developed respiratory failure requiring intubation within the first 2 h of life, with an escalation of invasive ventilation from conventional ventilation to high-frequency ventilation. Physical examination initially revealed increased work to breath, which improved with respiratory support. No crackles or wheezes were auscultated, and no obvious dysmorphic features were observed. The initial chest radiograph using Carestream (Carestream Health) revealed diffuse infiltrates in the bilateral lung fields (Figure 1), and he was treated for neonatal respiratory distress syndrome with five doses of surfactant (single dose: 100 mg/kg birth weight) and intravenous antibiotics (cefotaxime and gentamycin).

**Figure 2:**
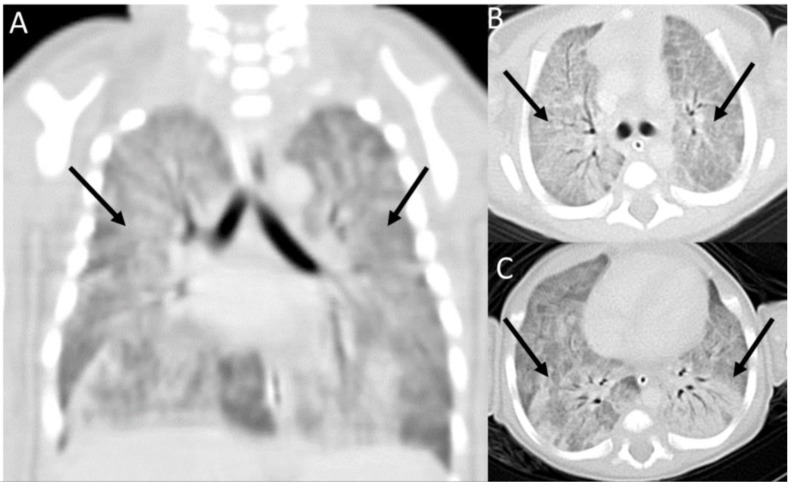
Chest-contrasted computed tomography (CT) findings of the patient: (**A**) Coronal CT chest image showed aeration of the large airways, with ground-glass material filling the distal airspaces and interlobular septal thickening (arrows). (**B**,**C**) Axial CT chest images showed extensive ground-glass opacification/consolidation of both lungs (arrows).

His initial echocardiogram evaluation on the second day of life showed normal heart structure and function, without evidence of persistent pulmonary hypertension. Serial chest radiographs demonstrated diffuse granular opacities. High-resolution chest computed tomography (HRCT) was performed on day 14 post-birth using a 16-sliced scanner (Siemens Healthcare, Erlangen, Germany) and showed bilateral diffuse ground-glass opacities with air bronchogram, and no evidence of cystic changes (Figure 2).

Despite receiving five doses of surfactant, mechanical ventilation, and antimicrobial therapies, the patient’s respiratory status did not improve, with a persistent ground-glass appearance upon chest X-ray. The pediatric pulmonology team was consulted, and they raised the suspicion of the inherited disorders of pulmonary surfactant dysfunction. Whole-exome sequencing (WES) was performed, and the neonate continued to receive respiratory support. Deoxyribonucleic acid (DNA) was extracted using a PerkinElmer Chemagic DNA CS200 DNA extraction kit (PerkinElmer, Waltham, MA, USA) with the Chemagic 360 instrument (PerkinElmer, Waltham, MA, USA). DNA quality was checked and quantified with PicoGreen reagent (ThermoFisher, Waltham, MA, USA) and an Enspire plate reader (PerkinElmer, Waltham, MA, USA). Whole-exome sequencing was performed on the genomic DNA using the Agilent SureSelect Clinical Research Exome v3 targeted sequence capture method to enrich for the exome following standard protocols [16]. Direct sequencing of the amplified captured regions was performed using 2 × 150 bp paired-end reads on NovaSeq 6000 Illumina next-generation sequencing (NGS) systems. The target region included the exon and 10 bp of the flanking intronic region. Primary data analysis was performed using the Illumina bcl2fastq converter v2.19. Secondary analysis was performed using the Illumina DRAGEN Bio-IT Platform v.3.4.12. Tertiary data analysis was performed using SnpEff v5.0 and PerkinElmer’s internal ODIN v.1.01 software. Alignment to the human reference genome (hg19) was performed, and annotated variants were identified in the targeted region.

The variants were evaluated by their frequency as reported in public databases such as the Genome Aggregation Database (gnomAD), Human Gene Mutation Database (HGMD), and ClinVar. Variant interpretations and classifications were performed using the American College of Medical Genetics (ACMG) standards and guidelines for the interpretation of sequence variants, and as per ClinGen guidelines on sequence variant interpretation. Copy number variants (CNVs) and absence of heterozygosity (AOH) analysis were assessed using NxClinical 5.1 software (BioDiscovery, El Segundo, CA, USA). CNVs were detected using the hidden Markov model-based fast adaptive state segmentation technique algorithm. The following logR cutoff values were used to analyze CNVs: high copy number calls expected to have >0.85, copy number gain between 0.35 and 0.85, copy number loss between −0.5 and −1.25, and high copy number loss <−1.25 [17]. CNV analysis was designed to detect deletions and duplications; in some instances, due to the size of the exons or other factors, not all CNVs may have been analyzed.

Throughout their hospital stay, the neonate continued to exhibit respiratory insufficiency and hypoxia and remained on respiratory support and tube-feeding. On day 70 post-birth, the WES results revealed a homozygous variant in the IDUA gene (c.46_57del; p.Ser16_Ala19del and p.Ser16_Ala19del), confirming a diagnosis of MPS I. This variant has been reported in compound heterozygosity with other IDUA variants in at least six individuals with MPS I [18,19,20,21,22], and it has also been reported as a homozygous disease-causing variant [2]. Enzymatic testing at a later stage showed IDUA deficiency; the patient’s result was 0.5 µmol/L/h (reference value >1.5 µmol/L/h).

**Figure 3:**
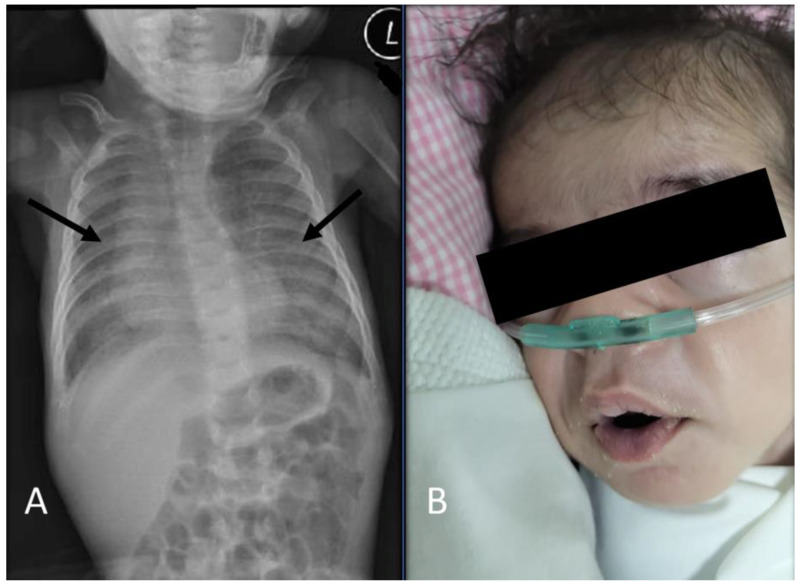
Chest radiography (CXR) (at 2 months) and the patient’s facial features at the age of 2 months: (**A**) CXR (at 2 months) revealed a persistent bilateral ground-glass appearance (arrows). (**B**) The patient at the age of 2 months showed mild dysmorphic facial features, including mild coarse facial features, a short and flat nose with wide nostrils, broad eyebrows with mild synophrys, and facial hypertrichosis. L, left.

Physical examination of the MPS I diagnosis (at two months old) revealed mild dysmorphic features, including mild coarse facial features, a short and flat nose with wide nostrils, broad eyebrows with mild synophrys, and facial hypertrichosis (Figure 3).

**Figure 4:**
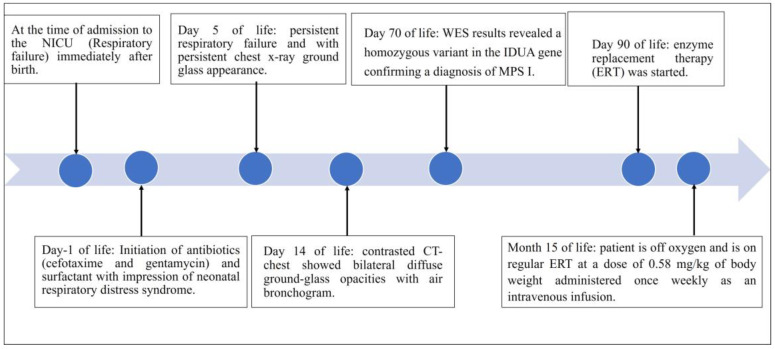
Timeline from the first presentation to the age of 15 months (12 months after the initiation of enzyme replacement therapy).

A plain chest X-ray revealed a persistent bilateral ground-glass appearance. An abdominal ultrasound revealed mild hepatosplenomegaly. The cranial ultrasound was normal, with no evidence of hydrocephalus. An echocardiography ultrasound system (iE33, Philips Medical Systems, Andover, MA, USA) at two months of age demonstrated normal heart function and structure without evidence of pulmonary hypertension. Due to the unavailability of enzyme replacement therapy (ERT) or hematopoietic stem cell transplantation, the patient was sent to a specialized center for further management. The patient is now 15 months old and has been successfully weaned off oxygen, only requiring oxygen during respiratory illnesses, and is on regular ERT at a dose of 0.58 mg/kg of body weight administered once weekly as an intravenous infusion (Figure 4). Figure 4 provides the patient’s diagnostic and therapeutic timelines.

## 3. Discussion

This case report described a genetically confirmed instance of MPS I in a late preterm baby with neonatal onset of interstitial lung disease. The necessity of considering MPS I as a potential cause in neonatal differential diagnosis needs to be considered during the presence of persistent respiratory insufficiency.

In neonates with diffuse lung disease, the cause is often attributed to aspiration, infection, and/or congenital heart disease. However, MPS I with interstitial lung disease as a primary presenting manifestation has not been extensively reported on [13,15]. In the current case, MPS I was diagnosed with the neonate showing interstitial lung disease, which is an uncommon feature of MPS I [2,9,10,11,22,23,24]. The official clinical guidelines of the American Thoracic Society (ATS) for evaluating childhood interstitial and diffuse lung disease (chILD) state that specialized consultations should be considered after excluding the common causes of diffuse lung diseases [25]. The ATS guidelines recommend echocardiography, controlled ventilation, HRCT, infant pulmonary function testing, bronchoscopy with bronchoalveolar lavage, genetic testing, and/or lung biopsy [25]. In the diagnosis of the current neonatal case workup, chILD was initiated due to an atypical presentation of the respiratory status and, importantly, a lack of response to multiple surfactant therapies. Hence, inherited pulmonary surfactant dysfunction disorders were highly suspected, which was further supported by persistent radiological findings, including a ground-glass appearance, which was all within the acceptable clinical guidelines of differential diagnosis [13,25].

Typically, MPS I is characterized by upper airway obstructive symptoms, restrictive and interstitial respiratory disease, and recurrent respiratory infections, complicating the severity of the clinical presentation [7]. However, early neonatal respiratory insufficiency is considered a rare presentation [13]. One study depicted two cases of MPS I-related interstitial lung disease with detailed electron microscopy findings, and the primary finding was membrane-bound GAG within interstitial cell lysosomes [5]. Although the current observation lacked electron microscopic observations of the GAG membrane deposition, radiographic findings, along with IDUA deficiency, proved beyond a doubt that MPS I was the underlying disease in the patient. Another study reported a similar neonatal manifestation as described in this case, except the patient was diagnosed with MPS II [9]. However, it should be noted that MPS II is diagnosed histologically with pulmonary interstitial glycogenosis correlating with classical MPS II clinical manifestations [9,10]. Taken together, these data show that the pulmonary interstitium, particularly cell lysosomes, is involved in different types of MPS and could cause severe interstitial lung disease early in life, with persistent hypoxemia and severe oxygen diffusion defects [9,10].

The causative defect of MPS I is a mutation of the IDUA gene, which causes enzyme deficiency, resulting in the accumulation of the two types of GAGs, heparan sulfate and dermatan sulfate, in different tissues and organs, which then causes the development of progressive multisystem pathologies [2,4,5,6,7,8,9,26,27]. Based on a review of previously published studies, there are >300 causative or associated IDUA gene mutations [4]. However, the understanding of the genotype–phenotype correlation is evolving and requires further multinational analysis [2,4]. Only two cases of MPS I with early neonatal lung involvement have been reported with biallelic IDUA disease-causing gene mutations (c.208C > T, p.Q70X, and c.1205G > A, p.W402X). [13]. The current patient had homozygous IDUA mutations (c.46_57del (p.Ser16_Ala19del)). This mutation was previously reported to cause early symptoms and progressive disease [16,17,19,22]. Our patient had low enzyme levels with overt dysmorphic features at the age of two months, which provided further evidence that this particular mutation was truly pathogenic with a unique presentation. Considering the results of WES in neonates with respiratory failure after excluding common etiologies is thus recommended.

The diagnosis of MPS I needs a constellation of clinical features, documented low IDUA enzyme activity, and evidence of two disease-causing mutations of the IDUA gene. Our patient had a rare MPS I manifestation with parenchymal lung involvement. In addition, the patient had documented low enzyme activity of IDUA and two disease-causing mutations of the IDUA gene. After confirming the MPS I diagnosis, the patient was started on ERT and recommended for HSCT at a specialty center. Prompt initiation of ERT provided clinical benefits, including both pulmonary and extrapulmonary manifestations [2,3,4,5,6,26]. In addition, based on the conclusion from a published MPS I registry, initiation of ERT in early infancy improved the neurodevelopmental outcomes and quality of life of the patient [7,11,12]. However, due to the rarity of the disease and the wide variability in clinical presentation and disease course, late diagnosis is common, with a poor prognosis and unwarranted complications [27].

## 4. Conclusions

This case report described the first documented case of a Saudi patient with MPS I that presented with early neonatal respiratory distress, initially diagnosed as inherited disorders of pulmonary surfactant dysfunction. Therefore, neonatal interstitial lung disease caused by MPS I should be considered in the differential diagnosis of patients with persistent respiratory manifestations not controlled by multiple surfactant therapies, and after ruling out the common etiologies of early neonatal pulmonary diseases such as respiratory distress syndrome and congenital pneumonia.

## Data Availability

The datasets used in this study are available at www.ncbi.nlm.nih.gov/clinvar/ under accession number SCV002023101.1 (accessed on 28 October 2022).
